# Suppression of radiation-induced migration of non-small cell lung cancer through inhibition of Nrf2-Notch Axis

**DOI:** 10.18632/oncotarget.16622

**Published:** 2017-03-28

**Authors:** Qiuyue Zhao, Aihong Mao, Ruoshui Guo, Liping Zhang, Jiawei Yan, Chao Sun, Jinzhou Tang, Yancheng Ye, Yanshan Zhang, Hong Zhang

**Affiliations:** ^1^ Department of Radiation Medicine, Institute of Modern Physics, Chinese Academy of Sciences, Lanzhou 730000, China; ^2^ Key Laboratory of Heavy Ion Radiation and Medicine of Chinese Academy of Sciences, Lanzhou 730000, China; ^3^ Gansu Wuwei Institute of Medical Sciences, Gansu Province, Wuwei 733000, China; ^4^ Key Laboratory of Basic Research on Heavy Ion Radiation Application in Medicine, Gansu Province, Lanzhou 730000, China; ^5^ University of Chinese Academy of Sciences, Beijing 100039, China; ^6^ Institute of Gansu Medical Science Research, Lanzhou 730000, China; ^7^ South China Normal University, Guangzhou 510642, China; ^8^ Northwest Normal University, Lanzhou 730000, China; ^9^ Lan Zhou University, Lanzhou 730000, China

**Keywords:** Nrf2, ionizing radiation, Notch1, EMT, NSCLC

## Abstract

Nuclear factor E2 related factor 2 (Nrf2) is a transcription factor that is associated with tumor growth and resistance to radiation. The canonical Notch signaling pathway is also crucial for maintaining non-small cell lung cancer (NSCLC). Aberrant Nrf2 and Notch signaling has repeatedly been showed to facilitate metastasis of NSCLC. Here, we show that radiation induce Nrf2 and Notch1 expression in NSCLC. Knockdown of Nrf2 enhanced radiosensitivity of NSCLC and reduced epithelial-to-mesenchymal transition. Importantly, we found that knockdown of Nrf2 dramatically decreased radiation-induced NSCLC invasion and significantly increased E-cadherin, but reduced N-cadherin and matrix metalloproteinase (MMP)-2/9 expression. We found that Notch1 knockdown also upregulated E-cadherin and suppressed N-cadherin expression. Nrf2 contributes to NSCLC cell metastatic properties and this inhibition correlated with reduced Notch1 expression. These results establish that Nrf2 and Notch1 downregulation synergistically inhibit radiation-induced migratory and invasive properties of NSCLC cells.

## INTRODUCTION

Lung cancer is the leading cause of cancer death worldwide, with non-small cell lung cancer (NSCLC) accounting for approximately 80% of all lung cancers [1]. Radiotherapy is routinely used for lung cancer treatment and is effective as a curative modality [2]. With the continuous improvement of diagnostic and therapeutic approaches, most patients with early stage NSCLC have been cured successfully. However, the 5-year survival rate for advanced stage NSCLC is as low as 15% [3], as these patients present with metastatic disease [4]. The mechanisms underlying lung cancer metastasis remain elusive. Thus, there is an urgent need to better understand the molecular mechanisms associated with radiation-induced carcinogenesis to improve radiotherapy treatments for lung cancer patients.

As an evolutionarily conserved signaling pathway, Notch signaling plays a critical role in cell fate determination, tissue patterning, cell proliferation and apoptosis [5, 6]. The Notch family of transmembrane proteins consists of four receptors (Notch1-4) and five ligands (Jagged1 and 2, Delta-like ligand (DLL) 1, 3 and 4) in humans. Following ligand binding, the Notch receptor is cleaved, releasing Notch intracellular domain (NICD) which can then translocate into the nucleus and act as a transcription factor to induce the target genes Hes-1, p21, Hey1 and others [7]. Recently, epithelial-to-mesenchymal transition (EMT) has been identified as one of the most important factors associated tumor metastasis. It is believed that abnormal Notch signaling is associated with tumorigenesis and metastasis and can induce EMT [8], Notch activation can promote migration and invasion of glioma cells [9], and reduced Notch1 decreases the invasion capacity of hepatocellular carcinoma and prostate cancer cells [5, 9]. Recent studies also demonstrate that Notch signaling is associated with tumor progression in lung cancer patients [10, 11]. In different types of lung carcinoma, Notch exhibits both tumor promoting and inhibiting functions. Notch1 is upregulated in 30% of primary human NSCLCs [12], and downregulation of Notch1 induces apoptosis [13], and reduces NSCLC invasion [14]. However, the role of Notch1 in radiation-induced carcinogenesis remains largely unknown.

Nuclear factor E2-related factor 2 (Nrf2) is a transcription factor that mediates a broad-based set of adaptive responses and regulates the expression of antioxidant and detoxification enzymes [15]. Recent studies found that Nrf2 plays a critical role in invasion [16, 17] and that Nrf2 may regulate Notch1. For example, expression levels of Notch1 and its target genes were downregulated in Nrf2^−^/^−^ MEF cells and upregulation of Nrf2 produced excess Notch signaling [18]. Moreover, Notch1 gene expression is regulated by Nrf2 in liver regeneration [19] and stem cell self-renewal [20]. Nrf2 enhances hematopoietic reconstitution by activating Notch signaling after total body irradiation [21]. However, on a mechanistic level, the role of Nrf2 in invasion of NSCLC after ionizing radiation (IR) remains unknown.

Our early observations found that downregulation of Nrf2 induced apoptosis of NSCLC via decreased Notch1 expression after X-ray irradiation [22]. Pursuant to these results, we here demonstrate that Nrf2 and Notch1 are upregulated by radiation and both Nrf2 and Notch1 signaling affect the invasiveness of NSCLC cells. Therefore, we hypothesized that Nrf2 could promote the migration and invasion of NSCLC cells with Notch1 signaling. In the present study, we found that the synergistic interaction between Nrf2 and Notch1 signaling in NSCLC cells plays a critical role in cell migration and invasion.

## RESULTS

### Nrf2 and Notch1 are upregulated in response to IR

**Figure 1:**
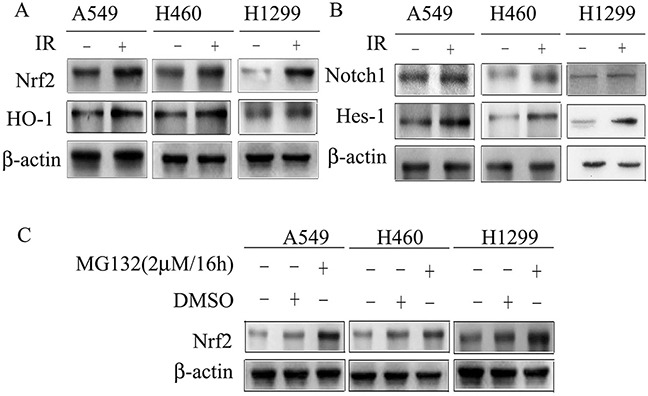
Cell lysates were subjected to Western blotting for the detection of Nrf2, HO-1, Notch1 and Hes-1 **(A)** X-rays irradiation induced Nrf2 and its target gene HO-1 expression in A549, H460 and H1299. **(B)** Notch1 and its target gene Hes-1 are induced in response to X-rays irradiation in A549, H460 and H1299. **(C)** MG132 induced Nrf2 expression in A549, H460 and H1299. β-actin was used as loading control. Results are from triplicate experiments.

Dysregulation of Nrf2 and Notch1 promotes survival and proliferation of cells after IR [8, 23]. To assess alterations in expression of Nrf2 and Notch1 in response to IR, we exposed A549, H460 and H1299 cells to 4Gy X-rays. Radiation treatment significantly increased the protein levels of Nrf2 and its target gene HO-1, as well as Notch1 and its target gene Hes-1, in cells (Figure 1A and 1B). In order to examine the degradation of Nrf2, cells were treated with the proteasome inhibitor MG132 (Figure 1C). Nrf2 degradation was significantly inhibited by MG132 in NSCLC cell lines. The results indicated that Nrf2 and Notch1 are involved in IR responses and Nrf2 is dependent on the proteasome-mediated protein degradation pathway.

### Nrf2 regulates Notch signaling in NSCLC

**Figure 2:**
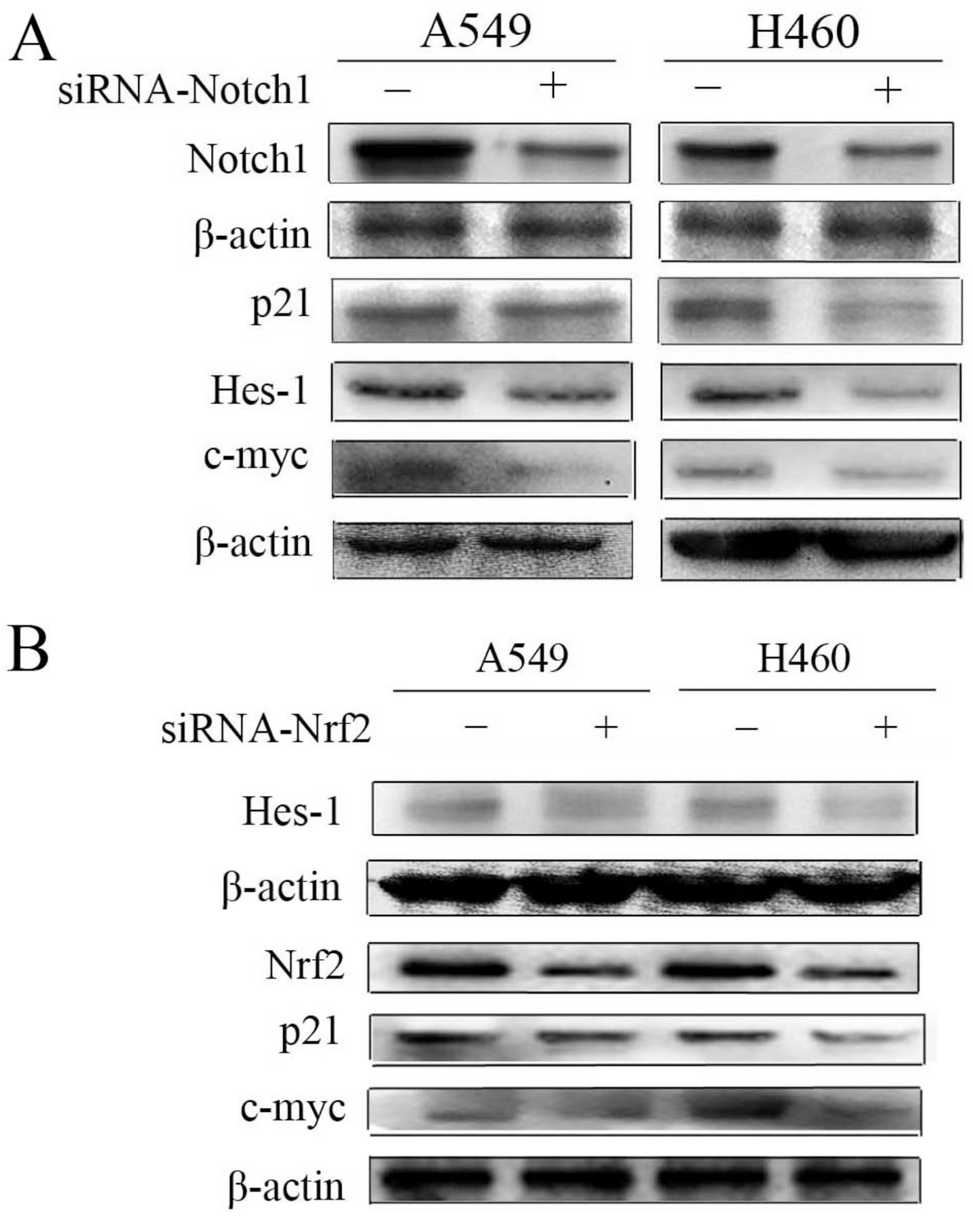
Down-regulation of Notch1 and Nrf2 in A549 and H460 cells **(A)** Immunoblot showing the levels of Notch1, p21, Hes-1, c-myc protein following transfection with non-specific or siRNA targeting Notch1 (siRNA-Notch1). **(B)** Hes-1, Nrf2, p21, c-myc protein levels following transfection with non-specific or siRNA targeting Nrf2 (siRNA-Nrf2) were monitored by western blot. β-actin served as normalization control. Results are from triplicate experiments.

Several studies have demonstrated that Nrf2 and Notch1 interact in specific contexts such as in liver regeneration, human airway basal stem cell self-renewal, osteoblastogenesis, and others [18, 20, 21, 24]. In order to verify the relevance of Nrf2 and Notch crosstalk in NSCLC, A549 and H460 cells were used. The results showed that Notch1 siRNA decreased the expression of Notch1 and its target genes p21, Hes-1 and c-myc (Figure 2A). Importantly, in addition to downregulation of Nrf2 by Nrf2 siRNA, protein levels of p21, Hes-1, and c-myc were also reduced (Figure 2B). These data suggest that Nrf2 regulates Notch signaling in A549 and H460 cells.

### Nrf2 knockdown sensitizes NSCLC to X-ray radiation

**Figure 3:**
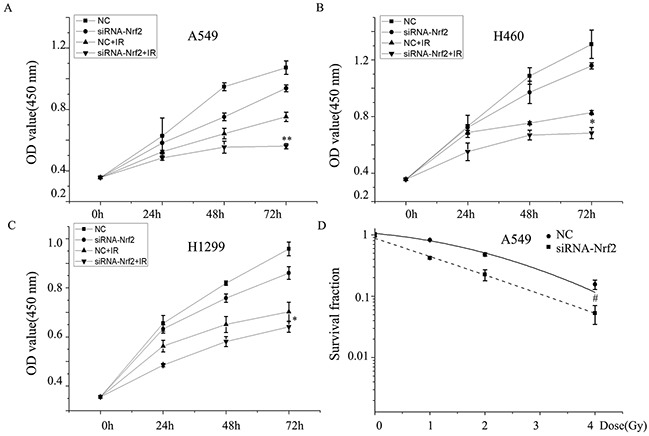
Knockdown of Nrf2 increased enhances radiosensitivity of NSCLC cells **(A)**, **(B)**, **(C)** cell proliferation and **(D)** colony formation of A549 cells transfected with siRNA-Nrf2, negative control (NC) or irradiation. Data are representative of at least three independent experiments. *P< 0.05, **P< 0.01 versus irradiation-treated groups, and #P< 0.05 versus NC group.

To assess the radiosensitizing effects of Nrf2 in NSCLC, the expression of Nrf2 was knocked down by transiently transfecting cells with siRNA against human Nrf2 (siRNA-Nrf2) and compared to NSCLC cells transfected with a scrambled non-specific siRNA (negative control, NC). The results showed that Nrf2-depleted cells exposed to X-ray irradiation exhibited decreased proliferation compared to irradiated cells (Figure 3A–3C). Clonogenic survival assay revealed that the survival fraction of A549 cells transfected with siRNA-Nrf2 was significantly lower after IR than that of cells transfected with negative control (Figure 3D). These data demonstrate that knockdown of Nrf2 decrease cell viability and suppressed cell proliferation after IR and suggests that Nrf2 knockdown sensitizes NSCLC cells to IR.

### Nrf2 knockdown inhibits the migration and invasion capacity of NSCLC

**Figure 4:**
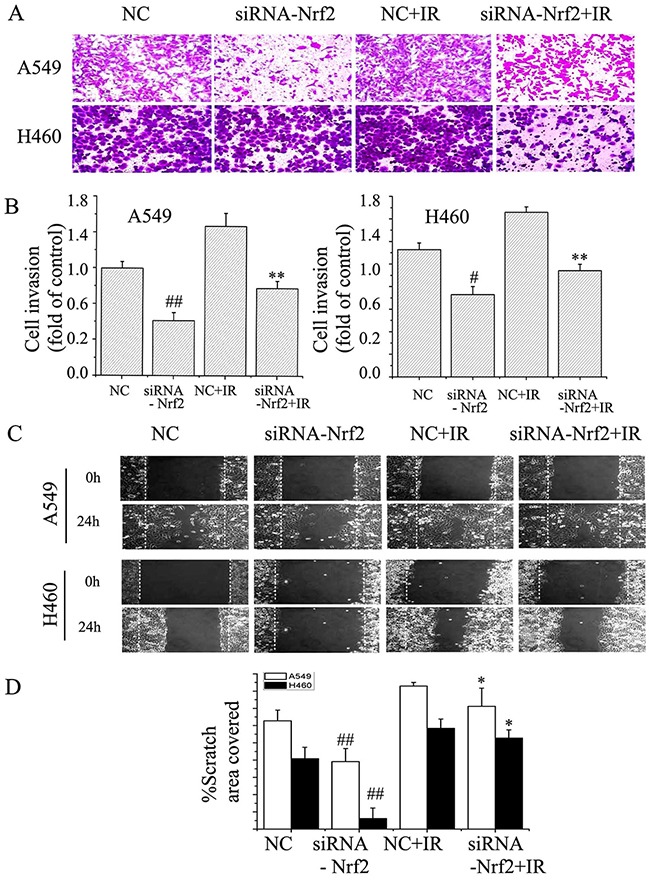
Suppression of Nrf2 attenuated EMT in NSCLC cells **(A)** and **(B)** Transwell invasion assay in A549 and H460 cells. The cells were then treated with or without the siNRA-Nrf2, or irradiation. The images (original magnification, ×200) were taken at 0 h and 24 h. **(C)** and **(D)** Scratch wound healing assay for A549 and H460 cells. The images (original magnification, ×100) were taken at 0 and 24 h after the wound was made. The dotted lines indicate the width of the wound at these two time points. The data are from triplicate experiments. #P< 0.05 and ##P< 0.01 versus NC group. *P< 0.05 and **P< 0.01 versus irradiation-treated groups.

It has been reported that Notch1 plays a key role in lung cancer cell migration and invasion [5, 25], which are both key cellular attributes necessary for metastasis. Because Nrf2 regulates Notch1 expression [19, 22, 26], we hypothesized that Nrf2 might be involved in NSCLC cell migration and invasion. Nrf2 knocked down cells were subjected to Transwell migration and scratch assays. The results showed that Nrf2 knockdown cells exhibited only marginal invasion through the extracellular matrix compared to control cells. NSCLC cells (A549 and H460) displayed a remarkable ability to invade 24 h post irradiation compared with non-irradiated cells (Figure 4A). Knockdown of Nrf2 inhibited radiation-induced cell invasion. Moreover, in H460 cells, treatment with Nrf2-siRNA also mildly suppressed radiation-induced NSCLC cell migration (Figure 4B). By 24 h after wounding, A549 cells had migrated into the wound, leading to almost complete gap closure after radiation. Similar effects on wound healing were noted in H460 cells (Figure 4C and 4D). Nrf2 knockdown significantly decreased in cell migration. The effects of Nrf2 knockdown suggest an essential role of Nrf2 in conferring migration and invasive properties to NSCLC cells.

### Knockdown of Nrf2 inhibits radiation-induced MMP-2 and MMP-9 in NSCLC

**Figure 5:**
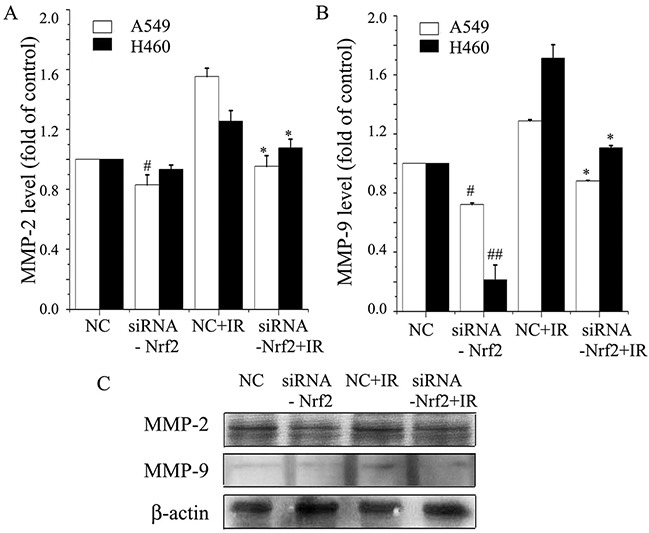
Knockdown of Nrf2 inhibits radiation-induced MMP-2 and MMP-9 expression levels in NSCLC **(A)** Briefly, A549 and H460 cells were transfected with siRNA-Nrf2, negative control (NC), or irradiation. ELISA assay analysis was used to determine MMP-2 levels. **(B)** MMP-9 levels were analyzed by ELISA assay in A549 and H460 cells. **(C)** Cell lysates were prepared and used for Western blot to determine the levels of MMP-2 and MMP-9 in A549 cells. β-actin was used as a control to confirm equal loading of cell lysates. Data are representative of at least three independent experiments. *P< 0.05 versus irradiation-treated groups, and #P< 0.05 and ##P< 0.01 versus NC group.

We analyzed the effect of reduced Nrf2 on A549 and H460 cells, two of the most aggressive lung cancer lines, for MMP-2 and MMP-9 expression. Enzyme-linked immunosorbent assay (ELISA)assays demonstrated that downregulation of Nrf2 inhibited MMP-2 and MMP-9 levels compared to negative control, with A549 and H460 cells expressing different base levels (Figure 5A). Furthermore, radiation alone augmented the expression level of these proteins. Treatment with Nrf2 knockdown plus radiation inhibited expression levels less than Nrf2 knockdown alone (Figure 5B). Western blot also showed that the levels of MMP-2 and MMP-9 in the irradiation group were elevated compared to the control group, and that knockdown of Nrf2 decreased MMP2 and MMP9 expression after irradiation (Figure 5C). These data suggested that reduced Nrf2 downregulates MMP-2 and MMP-9 expression and attenuated radiation-induced MMP-2 and MMP-9 expression.

### Downregulation of Nrf2 and Notch1 was associated with reduced cell migration

**Figure 6:**
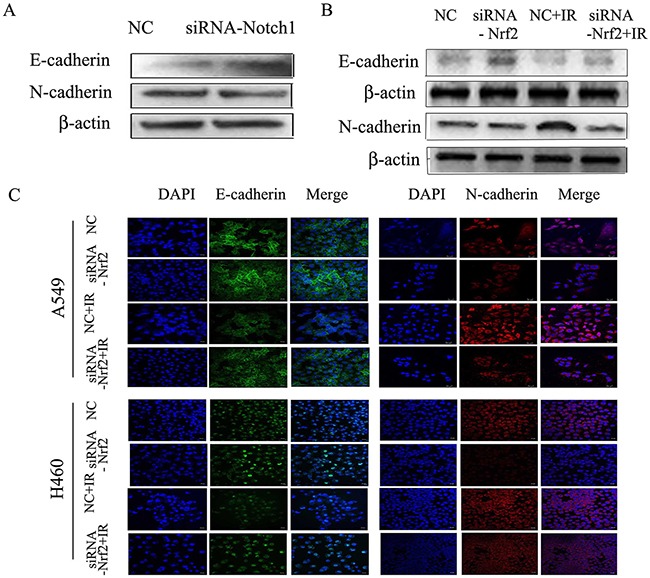
Down regulation of Notch1 or Nrf2 reduces radiation-induced EMT in NSCLC cells **(A)** A549 cells were transfected with siRNA- Notch1, or negative control (NC). Western blot results for E-cadherin and N-cadherin. **(B)** A549 cells were transfected with siRNA-Nrf2, negative control (NC) or irradiation. Western blot analysis of E-cadherin and N-cadherin. β-actin served as normalization control. **(C)** Immunofluorescence staining for E-cadherin and N-cadherin in A549 and H460 cells, Alexa Fluor 488-conjugated secondary antibody (green), Alexa Fluor 647-conjugated secondary antibody (red) and Nuclei were counterstained with DAPI (blue). Images were captured by confocal microscopy and merged. Bar=20 μm. Results are from triplicate experiments.

E-cadherin localizes to cell-cell junctions, and the expression of N-cadherin is associated with a more migratory and invasive phenotype. To further confirm the role Nrf2 in radiation-induced invasion, we assessed the expression of E-cadherin and N-cadherin. The results showed that Notch1 siRNA increased E-cadherin expression and decreased the expression of N-cadherin (Figure 6A). After treatment with siRNA-Nrf2, the expression of E-cadherin was enhanced compared to the control group, but the expression of N-cadherin was reduced. However, after irradiation, E-cadherin expression was reduced and the expression of N-cadherin was enhanced compared to negative control group. Reduction of Nrf2 reversed the radiation effect, as determined by both immunoblotting and immunofluorescence (Figure 6B and 6C). These data suggested that the role of Notch1 and Nrf2 in intercellular junctions involves regulation of E-cadherin and N-cadherin expression.

## DISCUSSION

In this study, we showed that IR induces Nrf2 activation, MMP-2/9 expression, N-cadherin expression and apoptosis, but reduces E-cadherin and subsequently contributes to NSCLC metastasis in an Nrf2-related mechanism. Moreover, Nrf2 silencing induced the downregulation of MMP-2/9 and N-cadherin, which is in part associated with suppression of the Notch1-dependent signaling pathway. These findings show that Nrf2 might act as a therapeutic target in the inhibition of cancer progression and provide a novel mechanistic insight into the potential drivers of NSCLC invasion and metastasis.

NSCLC is the most common cancer worldwide and is associated with high mortality because of its progression from delimited growth to invasive and metastatic growth, making complete surgical resection impossible. Nrf2 plays a pivotal role in endogenous protection against oxidative stress. However, several studies verified Nrf2 and its target genes are over expressed in NSCLC and lung cancer patients, giving cancer cells an advantage for survival and progression [27, 28]. Keap1 insertion and deletion mutations were found in 10 of 54 NSCLC patients, 6 of 12 cells line [29] and a high incidence of Keap1 somatic mutations was also found in 65 Japanese patients with lung cancer [30]. Somatic mutations in Keap1 constitutively activate Nrf2 expression. Furthermore, Nrf2 is up-regulated in resistant cancer cells and is thought to be responsible for acquired radio-resistance. Our previous studies suggest that inhibition of Nrf2 induces apoptosis-related protein expression and sensitizes NSCLC through Notch signaling [22]. Because there are limited reports about Nrf2-Notch1 interactions in NSCLC, we focused our attention on a potential role for Nrf2-Notch1 in NSCLC metastasis in this research.

Radiotherapy is critical to NSCLC, and consistent with a previous study, we found that IR increased Nrf2 protein and mRNA levels in A549 and H460 cells. It has been reported that A549 cells contain a Keap1 point mutation in which Gly is changed to Cys at amino acid 333 in the KR1 domain and H460 cells have a Asp to His substitution at amino acid 236 in the IVR domain [28], while H1299 cells express wild- type Keap1 [31], which may explain the different levels of Nrf2 in these cells. Irradiation activates the Nrf2 response and increases radioresistance of NSCLC. Our results revealed that knockdown of Nrf2 enhanced the apoptotic rate, increased the production of ROS, and decreased the survival in A549 and H460 cells. Several studies have investigated the promotion of cell metastasis by irradiation in many cancers. Radiation increases tumor invasion and metastasis via increased TGF-β1 expression [32, 33]. Furthermore, irradiated cells have been shown to acquire a more mesenchymal-like morphology [34]. In accordance with this finding, our results indicated that NSCLC invasion and metastasis increased after X-ray irradiation.

Several studies have shown that antioxidants can promoted cancer initiation and progression [35, 36]. Constitutive activation of Nrf2 in lung cancer cells promotes tumorigenicity and reduced Nrf2 expression inhibits tumor growth [37, 38]. Inhibition of Nrf2 also represses tumor metastasis and invasion [16] and impairs osteopontin-induced migration [39]. A recent investigation revealed that knockdown of Nrf2 attenuated naturally occurring tumor metastasis as well as metastasis induced by hypoglycemic dipeptidyl peptidase-4 inhibitors [40]. Loss of Nrf2 also reversed propofol-induced invasion [41]. Nrf2 target genes play critical roles in tumor metastasis. HO-1 participates in maintaining cellular homeostasis and is a main Nrf2 target gene [42]. It has been reported that lung cancer patients with high HO-1 levels had reduced overall survival rates compared with the patients with lower HO-1 tumor expression levels [43]. Furthermore, in lung cancer tumor samples, HO-1 was elevated about 4.7 fold compared to normal tissues [44]. A growing body of evidence indicates that inhibition of HO-1 inhibits the occurrence of metastasis [45–47] and suppresses MMPs [44]. NQO1 is another gene which protects against oxidative stress and can be induced by Nrf2. Higher NQO1 levels have been found in tumors with nodal metastases compared to tumors without metastases [48]. GCLM is an important target of Nrf2, a contributor to GSH synthesis, inhibition of this antioxidant diminishes cancer initiation [35]. Our data are in agreement with recent studies that demonstrated knockdown of Nrf2 reverses radiation-induced metastasis of NSCLC. However, an investigation reported that loss of Nrf2 enhances cellular plasticity and motility through TGF-β/Smad signaling [49] or increases tumorigenesis via elevated ROS levels [50]. We hypothesized that the differences in metastasis observed by this investigator and the work here may be attributed to different experimental approaches.

Accumulating data have demonstrated that Notch1 plays a critical role in cell fate decisions through cell-cell communication. Clinical data revealed that activated Notch1 was found in 30% of NSCLC cases [12] and Notch1 activating mutations have been described as a common event in NSCLC [51]. Moreover, activated Notch1 inhibits apoptosis and enhances radioresistance [52]. While, reduced Notch1 expression can induce radiosensitization in NSCLC cells. The invasion behavior of malignant cells is often accompanied by the loss of adhesion molecules, such as E-cadherin, and upregulated expression of mesenchymal markers, such as N-cadherin. Downregulation of Notch1 alleviated radiation-induced EMT [53]. It has been reported that expression of Notch1 downstream transcripts correlates with EMT [54]. This is consistent with previous findings where knockdown of Notch1 downregulated Notch1 and its target genes Hes-1, p21, c-myc. Notch1-siRNA also enhanced E-cadherin expression, but decreased N-cadherin and MMP-2/9 expression. Together, these results revealed that Notch1 could reduce EMT in NSCLC. It has been noted that Notch1 seems to play a prominent role in NSCLC migration and invasion. In the present study, we demonstrated that Hes-1, p21 and c-myc are downregulated by both Nrf2 siRNA and knockdown of Notch1. Notch1 mainly participates in cell-to-cell communication, thus the attenuated radiation-induced invasion and migration effects following inhibition of Nrf2 signaling may have resulted from downregulated Notch1 in NSCLC cells.

In summary, we have demonstrated that knockdown of Nrf2 is related to downregulation of Notch1, and the ability of Notch1 inhibition to block radiation-induced migration and invasion suggests synergistic effects between Nrf2 and Notch1. The roles of the Nrf2-Notch1 interactions in impeding tumor metastasis need to be explored more deeply. The findings of this present study might help to guide the development of potential therapeutic targets for the prevention of NSCLC cell metastasis.

## MATERIALS AND METHODS

### Cell culture and irradiation treatment

Human non-small cell lung cancer cells (A549, NCI-H460 (H460), NCI-H1299 (H1299)) were grown in RPMI-1640 (Gibco Life Technologies, USA) medium supplemented with 10% (v/v) fetal bovine serum (FBS) (Hyclone, GE Healthcare Life Sciences, USA) and incubated at 37°C in a humidified 5% CO_2_ atmosphere.

Cells were irradiated with X-rays using Faxitron RX-650 (Faxitron Bioptics, LLC, USA). The dose rates were 0.674 Gy/min.

### RNA interference of Nrf2

SiRNA against Nrf2, Notch1 and non-targeting negative control siRNA were purchased from Santa Cruz Biotechnology (Santa Cruz, CA). Nrf2 siRNA (h) is a pool of three different siRNA duplexes: (1) sense: 5′-GCAUGCUACGUGAUGAAGAtt-3′, antisense: 5′-UCUUCAUCACGUAGCAUGCtt-3′, (2) sense: 5′-CUCCUACUGUGAUGUGAAAtt-3′, antisense: 5′- UUUCACAUCACAGUAGGAGtt-3′ and (3) sense: 5′-GUGUCAGUAUGUUGAAUCAtt-3′, antisense: 5′- UGAUUCAACAUACUGACACtt-3′. Notch 1 siRNA (m) also contains three different siRNA duplexes: sense: (1) 5′- CCCUUUGAGUCUUCAUACAtt-3′, antisense: 5′- UGUAUGAAGACUCAAAGGGtt-3′, (2) sense: 5′-GAAGGUGUAUACUGUGAAAtt-3′, antisense: 5′-UUUCACAGUAUACACCUUCtt-3′ and (3) sense: 5′-CAAGGAGUCUGAAGACUAUtt-3′, antisense: 5′-AUAGUCUUCAGACUCCUUGtt-3′. Cells were transfected with Nrf2, Notch1 or scrambled non-specific siRNAs. Cells were plated onto new plates 1 day before transfection with Lipofectamine 2000 (Invitrogen Life Technologies, USA) following the manufacturer's instructions. The serum-free medium was replaced with new culture medium 6 h after transfection. The cells used in the following experiments were transfected for 48 h.

### Cell proliferation assays

Cell proliferation rate was determined using Cell Counting Kit-8 (CCK-8, Dojindo, Japan) according to the manufacturer's protocol. Briefly, 2000 cells per well were seeded in duplicate in 96-well plates and incubated for 24, 48 and 72 h at 37°C in a humidified incubator (six wells per group, total). Then, cells were incubated with CCK-8 solution (10μl per well) for 1.5 h and absorbance was measured at 450 nm using a multimode reader (Thermo Varioskan Flash 3001).

### Colony formation assay

After irradiation, cells were washed with PBS, trypsinized and resuspended in RPMI-1640 medium supplemented with 10% FBS. An appropriate number of cells were plated into each 60 mm dish and three parallel dishes were scored for each treatment. After incubating for 14 days post-irradiation, cells were fixed with 100% methanol for 30 min and stained with 0.5% crystal violet. Colonies with more than 50 remaining cells were counted.

### Matrigel invasion assays and wound healing migration

Cells (1×10^6^) were resuspended in 400μl of serum-free medium and seeded in Matrigel (BD Biosciences, USA) coated Transwell upper chambers (Millipore, USA) with 8.0 μm polycarbonate filter inserts in 24-well plates, while the bottom chambers were filled with 600 μl complete medium. After incubation for 24 h, non-migrated cells and the matrigel were scraped using a cotton swab. The bottom side of the membrane was fixed with ethanol and stained with Giemsa. The Transwell chambers were washed three times with PBS. Images of migrated cells were obtained using a microscope (Carl Zeiss, Germany).

Cells were cultured on 35-mm culture dishes to confluence. Scratch wounds were created on monolayers using a sterile 200 μl micropipette tip, and cells were then washed twice with fresh medium. Cells migrated into the wounded area, and photographs were taken immediately 0 h and 24 h later.

### ELISA

MMP2 and MMP9 were analyzed by ELISA (Excell Bio, China) according to the manufacturer's protocol. The optical density (OD) values were detected using a microplate reader (Tecan Infinite M200, Swiss) at a wavelength of 450 nm. The absolute value of MMP2 and MMP9 was calculated according to the standard curve.

### Western blot analysis

Protein from cells were mixed with RIPA buffer (Beyotime, China) containing 1mM PMSF. Protein extracts were obtained using a total and nuclear protein extraction kit (Thermo, USA). Equal amounts of protein were loaded onto 10% SDS-PAGE and proteins were transferred to PVDF (Roche). The membranes were then blocked with 0.05% Tween and 5% bovine serum albumin (BSA) (BBI life sciences Corporation, Canada) in Tris-buffered saline for 2 h at room temperature and incubated overnight at 4°C with primary antibodies against Nrf2, Notch1, HO-1, p21, Hes-1, c-myc, MMP-2, MMP-9, E-cadherin, N-cadherin (Abcam, USA). The preparative membranes were incubated with appropriate secondary antibodies conjugated to HRP. The Immunological complexes were visualized with electrochemiluminescence (Millipore, Germany). Band intensities were analyzed using Image J software.

### Immunofluorescence staining

Cells were pretreated for 24 h with siRNA targeting Nrf2 and then exposed to irradiation. Cells were cultured on coverslips and fixed in 4% paraformaldehyde for 20 min at room temperature. Cells were rinsed three times with PBS and incubated with 0.3% Triton X-100 for 10 min. The cells were washed three times and blocked with 5% BSA in PBS for 1 h. Primary anti E-cadherin and anti N-cadherin antibody (Abcam, USA) were added and incubated overnight at 4°C. After three washes with PBS, cells were incubated for 1 h at room temperature with the appropriate secondary antibody. After PBS washes, the slides were incubated with 0.5 mg/ml DAPI (4′,6′-diamidino-2-phenylindole) at room temperature for 5 min. All images were observed under a confocal microscope equipped with a digital camera (LSM700; Carl Zeiss).

### Statistical analysis

Significant differences in means between two samples were analyzed using Student's t tests. *P* < 0.05 was considered significant. All graphs show the means ± standard error from at least three independent experiments.
